# Co-occurrence of amyotrophic lateral sclerosis and Leber’s hereditary optic neuropathy: is mitochondrial dysfunction a modifier*?*

**DOI:** 10.1007/s00415-022-11355-w

**Published:** 2022-09-06

**Authors:** Giulia Amore, Veria Vacchiano, Chiara La Morgia, Maria L. Valentino, Leonardo Caporali, Claudio Fiorini, Danara Ormanbekova, Fabrizio Salvi, Anna Bartoletti-Stella, Sabina Capellari, Rocco Liguori, Valerio Carelli

**Affiliations:** 1 Department of Biomedical and Neuromotor Sciences (DIBINEM), University of Bologna, Bologna, Italy; 2 IRCCS Istituto delle Scienze Neurologiche di Bologna, UOC Clinica Neurologica, Bologna, Italy; 3 IRCCS Istituto delle Scienze Neurologiche di Bologna, Programma di Neurogenetica, Bologna, Italy; 4 IRCCS Istituto delle Scienze Neurologiche di Bologna, Programma SLA Atassia Amiloidosi e Miastenia, Bologna, Italy; 5 Department of Experimental, Diagnostic and Specialty Medicine, DIMES University of Bologna, Bologna, Italy; 6 Bellaria Hospital, Via Altura 3, 40139 Bologna, Italy

Dear Sirs,

Amyotrophic lateral sclerosis (ALS) is a progressive neurodegenerative disorder causing selective loss of motor neurons in brain motor cortex and spinal cord in which muscle denervation and atrophy are associated with pyramidal involvement, typically spreading to contiguous muscular regions and leading eventually to a fatal paralysis. The pathogenic role of mitochondrial dysfunction in ALS has been investigated as there is evidence of morphological and biochemical mitochondrial abnormalities, both in patient tissues and animal models, suggesting their significant contribution to motor neurons degeneration since early phases of ALS [1]. Here, we report two ALS cases carrying the m.11778A > G/*MT-ND4* (R340H) mitochondrial DNA (mtDNA) mutation pathogenic for Leber’s hereditary optic neuropathy (LHON), a maternally inherited disease due to mtDNA mutations affecting complex I function and usually leading to isolated optic atrophy [2].

Both patients had positive family history for LHON and were followed on a regular basis. Routine neuro-ophthalmological evaluation included visual acuity, fundus oculi pictures, Optical Coherence Tomography (OCT Stratus, Zeiss) and computerized visual fields (VF Humphrey, Zeiss). LHON mutations were investigated by complete mtDNA sequencing, as previously reported [3]. ALS diagnosis was established using Awajii criteria [4]. The clinical and instrumental workout included: neurological examination, electromyography, and brain MRI. A complete blood exam screening was performed to exclude autoimmune and paraneoplastic mimics. Patient 2 also performed muscle biopsy on the left biceps. A targeted multigenic Next Generation Sequencing (NGS) panel for neurodegenerative disorders was carried out, including ALS-associated genes (list of genes available upon request), in both patients [5]. We compared age at onset and disease duration of our patients with an extensive dataset from our local ALS registry (BoReALS registry), collected from 2010 to 2019 at the Bellaria Hospital (Bologna, Italy), composed of 330 ALS patients (55 with a genetic diagnosis in one of the major ALS-associated genes *FUS, SOD1, TDP43* and *C9orf72*), 232 of which with spinal and 98 with bulbar onset [5]. This study was performed in accordance with the ethical standards of our institution and with the 1964 Helsinki declaration.

Case 1 (female) presented subacute bilateral vision loss at age 26. She carried the homoplasmic m.11778A > G/*MT-ND4* mtDNA mutation associated with LHON and other maternal relatives were similarly affected (pedigree on Fig. 1A). Neuro-ophthalmological follow-ups showed a severe bilateral optic atrophy at fundus examination (Fig. 1C) and OCT, with counting fingers as residual visual acuity. At age 73, she developed progressive hyposthenia of the right lower limb, spreading in 7 months to homo and contralateral muscular regions and in 10 months to bulbar region (timeline in Fig. 1A). Diagnosis of clinically definite ALS was made 11 months after onset. Neurological workout results are shown in Fig. 1B and brain MRI in Fig. 1D. She died 18 months after onset because of respiratory failure.

**Fig. 1:**
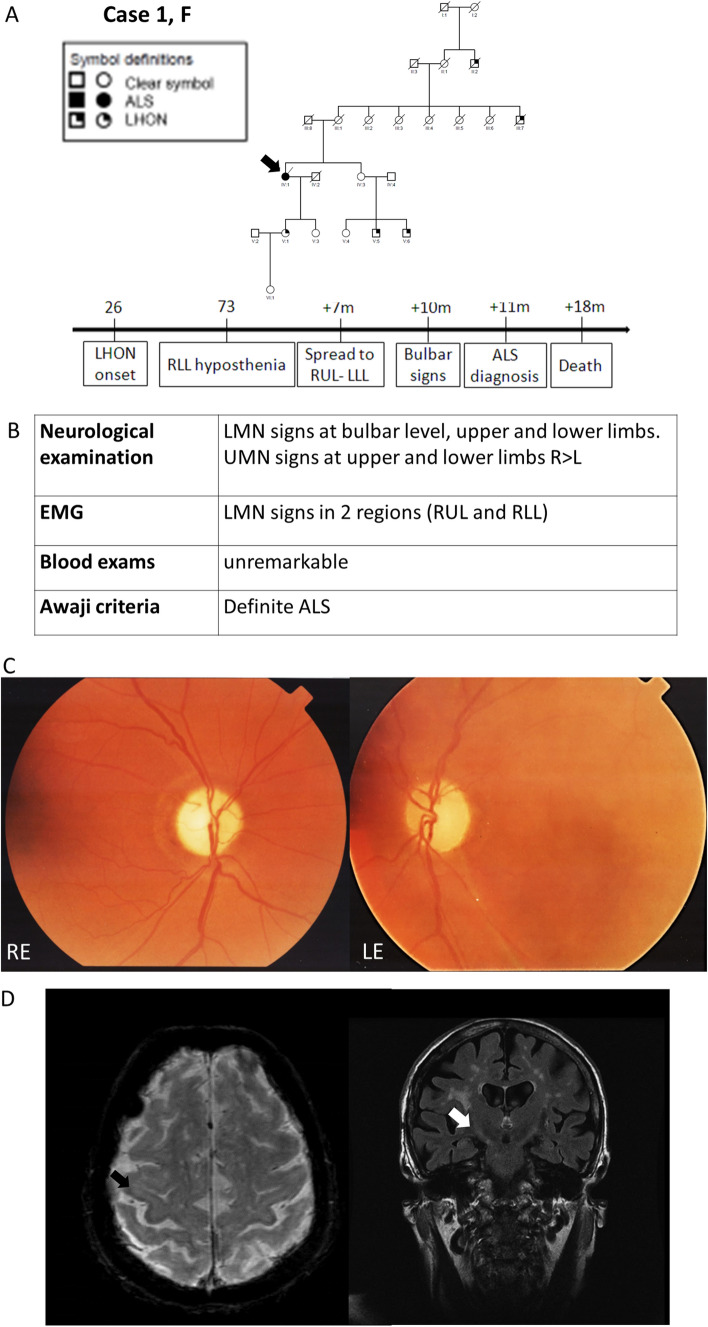
Case 1. Panel **A** Pedigree and clinical history, the proband is pointed by arrow. Panel **B** Results of the neurological workout. Panel **C** Fundus imaging showing bilateral optic atrophy with prevalent temporal pallor (right eye on the left, left eye on the right). Panel **D** Brain MRI. On the left, axial GE T2* sequence showing bilateral hypointensity of the motor cortex due to deposition of ferromagnetic material (black arrow). On the right, coronal FLAIR T2-weighted sequence displaying hyperintensity of the cortico-bulbar tract (white arrow). Acronyms: *RLL* Right Lower Limb, *RUL* Right Upper Limb, *LLL* Left Lower Limb, *LUL* Left Upper Limb, *LMN* Lower Motor Neuron, *UMN* Upper Motor Neuron, *RE* Right Eye; *LE* Left Eye, *EMG* electromyography

Case 2 (female) was an unaffected carrier of the m.11778A > G/*MT-ND4* mutation, with a nephew affected by LHON (pedigree on Fig. 2A). At neuro-ophthalmological evaluation there was no evidence of optic atrophy and OCT was normal except for a slight increase of retinal nerve fiber layer thickness in the temporal quadrant, compatible with the LHON mutation carrier status (Fig. 2C). At age 74, she presented progressive dysphagia and dysarthria, leading in 10 months to positioning of percutaneous endoscopic gastrostomy. In 12 months after onset, she developed dyspnea and proximal hyposthenia (timeline in Fig. 2A), meeting the diagnostic criteria for definite ALS (clinical-instrumental results are shown in Fig. 2B and D). She rapidly worsened and died 22 months after onset for respiratory failure. Muscle biopsy (Fig. 2E) showed evidence of subsarcolemmal proliferation of mitochondria and two ragged-red-fibers (RRF), with scattered cytochrome-c-oxidase (COX) negative fibers and multiple atrophic fibers with a neurogenic pattern.

**Fig. 2:**
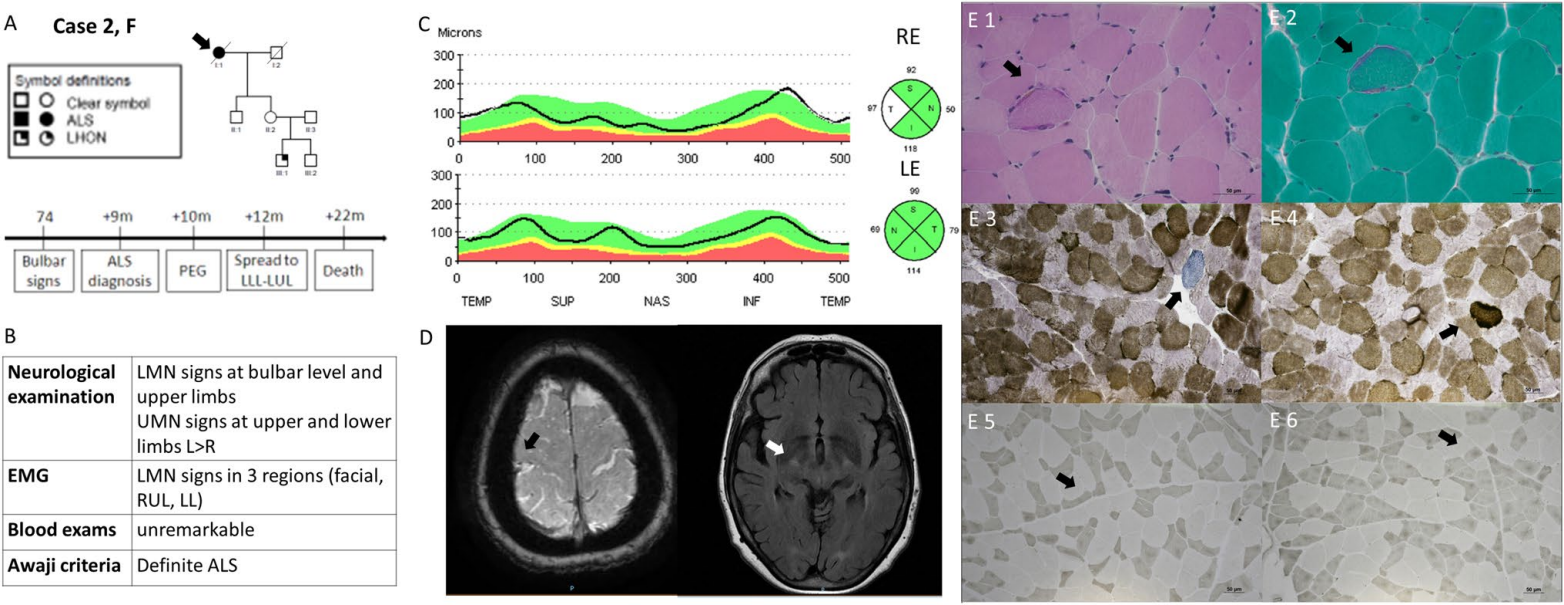
Case 2. Panel **A** Pedigree and clinical history, the proband is pointed by arrow. Panel **B** Results of the neurological workout. Panel **C** OCT showing normal optic nerve appearance with slight increase of RNFL thickness in temporal sector, right eye (up) more than left eye (down). Panel **D** Brain MRI. On the left, axial GE T2* sequence showing atrophy and hypointensity of the right motor cortex (black arrow). On the right, axial FLAIR T2-weighted sequence displaying hyperintensity of the cortico-spinal bundle in the internal capsule (white arrow). Panel **E** Muscle biopsy. A ragged red fiber is shown on Gomori trichrome stain (E.1) and hematoxylin-eosin stain (E.2) indicating a peripheral accumulation of abnormal mitochondria. With the combined cytochrome oxidase (COX)/succinic dehydrogenase (SDH) staining scattered fibers devoided of COX activity were observed (E.3) together with fibers showing subsarcolemmal mitochondrial proliferation (E.4). The adenosine triphosphatase (ATPase) staining, ph 9.4, showed neurogenic changes with angulated, atrophic fibers (E.5) and fiber type grouping (E.6). Acronyms: *RLL* Right Lower Limb, *RUL* Right Upper Limb, *LLL* Left Lower Limb, *LUL* Left Upper Limb, *LMN* Lower Motor Neuron, *UMN* Upper Motor Neuron, *RE* Right Eye; *LE* Left Eye, *OCT* Optic Coherence Tomography, *RNFL* Retinal Nerve Fiber Layer, *EMG* electromyography

The complete mtDNA sequencing, beside the LHON-associated mutation (homoplasmic m.11778A > G/*MT-ND4* in both cases, on U6a5 and H1b haplogroup, respectively), disclosed in case 1 a second homoplasmic m.10192C > T/*MT-ND3* (p.Ser45Phe) variant, which is predicted as possibly pathogenic and affects the same codon of a pathogenic mutation (m.10191 T > C; p. Ser45Pro) associated with a spectrum of phenotypes [6], but leading to a different amino acid change. This variant is rarely observed in the general population (93 cases in GenBank) indicating that is a polymorphism. Case 2 mtDNA was unremarkable except for the m.11778A > G/*MT-ND4* mutation.

The NGS panel revealed variants of interest in both patients. Case 1 carried the c.142A > C, p.Lys48Gln heterozygous variant in the *CHMP2B* gene (OMIM #609512, phenotype: Frontotemporal dementia and/or amyotrophic lateral sclerosis 7, autosomal dominant). The variant is novel and is classified as of uncertain significance with minor pathogenic evidence according to the ACMG classification. Case 2 carried the c.587C > T p.Pro196Leu heterozygous variant in the *PINK1* gene (#OMIM 608309, phenotype: Parkinson’s disease 6, early onset, autosomal recessive). This variant was reported in a patient with sporadic Parkinson’s disease and is classified as of uncertain significance [7].

We compared age at onset and disease duration of our two patients with demographic data from the BoReALS registry [5]. Median age of onset and disease duration of all 330 ALS patients were 63 years (range 27–87) and 35 months (range 4–169), respectively. Excluding the 55 cases with a genetic diagnosis in the most frequent ALS genes, which may have an earlier onset, the median age of onset was 64 years and the disease duration 38 months. Patients with spinal form had a median age at onset of 62 years (range 33–86) and 41 months (range 4–133) of disease duration. In patients with bulbar onset, median age at onset was 67 years (range 37–87) and the disease duration 29 months (range 5–119). Thus, in comparison, both our cases presented a delayed onset of ALS at 73–74 years (10 years later even excluding the genetic cases), and shortened disease duration of 20 ± 2 months (about 15–18 months shorter).

We reported the unique association of ALS co-occurring with LHON mutations, one of the probands being also affected with optic atrophy. Notably, both cases were remarkable for a relative late onset of ALS and aggressive course of disease. We consider the co-occurrence of ALS and LHON in these two patients as coincidental. Nevertheless, we argue that intrinsic mitochondrial dysfunction due to the LHON mutation may have acted as modifying factor in the natural history of ALS, in light of the rapid disease course observed in both patients. Thus, we conclude that mtDNA should be systematically re-valuated as genetic modifier in ALS.

A wide body of evidence documents mitochondrial dysfunction in patients with either sporadic or genetic forms of ALS [8]. In addition to the metabolic defect, alterations in mitochondrial dynamics and transport have been observed either in fibroblasts or neurons from patients as well as in animal models mutant for *SOD1*, *FUS* or *TDP-43* genes [9]. Increased proportion of COX-negative and RRF fibres, hallmarks of mitochondrial dysfunction, have been described in skeletal muscle of ALS patients [10], along with the typical pattern of denervation with clustered atrophic fibres and fibre type-grouping. The muscle biopsy of our patient (case 2) documented indeed a few RRFs, which are unusual in LHON carriers, normally displaying only modest subsarcolemmal compensatory proliferation of mitochondria [11].

The role of mtDNA mutations in ALS remains poorly investigated. Pathogenic mtDNA mutations have been found in ALS phenocopies with a primary mitochondrial disorder [12] and, more recently, mutations in nuclear genes, such as *CHCHD10*, have been associated with ALS and mitochondrial myopathy with accumulation of mtDNA multiple deletions [13]. Case 1 presented an adjunctive mtDNA variant and both patients carried variants of uncertain significance in nuclear genes, which could contribute more directly to ALS pathogenesis, possibly worsening mitochondrial dysfunction. Indeed, deficits in mitochondrial activity have been shown in induced pluripotent stem cells (iPSC)-derived neurons from CHMP2B^Intron5^ mutated patients [14]. Despite *PINK1* is not an ALS causative gene, its altered expression at both mRNA and protein levels have been identified in ALS patients’ muscle [15]. This scenario of multiple variants, in nuclear and mitochondrial genomes, possibly contributing to multilayered mitochondrial dysfunction, highlights the complexities of the genetic background in sporadic ALS. Recently, one of the largest studies ever on ALS pointed to the burden of multiple risk factors disclosed in the nuclear genome, missing however to consider the impact of mtDNA variation [16].

LHON mutations rarely present with other neurological symptoms, defining the so-called “LHON-plus” phenotype [2]. Amongst these, “Harding’s disease” is the debated association of LHON with multiple sclerosis, most likely due to chance again, but for which a reciprocal modifying effect leading to a distinct and more severe phenotype has been similarly discussed [17]. Certainly, most pathogenic mechanisms reported in LHON, including increased ROS production, abnormal quality control and increased propensity to apoptosis [18], may all well contribute to motor neuron degeneration, as well as dysregulation of microglia and inflammatory phenotypes, all key elements implicated in ALS, potentially contributed by mitochondrial dysfunction as recently discussed [19, 20].

All considered, two main conclusions are drawn from this study: first, given the rarity of the two disorders, 3/100,000 for LHON [17], 6/100,000 for ALS [21], most probably the two disorders co-occurred by chance, as already suggested for “Harding’s disease”; second, we documented a modified phenotype of the LHON-ALS association compared to classic ALS, again recalling LHON-MS. If this is the case, we wonder if these patients may benefit from a specific antioxidant therapy, such as idebenone, approved for acute LHON by EMA [22].

To conclude, our limited observations on two LHON-ALS cases are far from being definitive but highlight the contributory role that mitochondrial function and its small genome may play in ALS. Our observations should prompt large-scale studies designed to test the contribution as ALS risk factors of genetic variants in both mtDNA and nuclear genes encoding mitochondrial proteins (about 1500), as well as their possible role as disease modifiers.

## Data Availability

The authors take full responsibility for the data, the analysis, and interpretation of the research, and they have full access to all of the data.
